# Effect on Gait Speed, Balance, Motor Symptom Rating, and Quality of Life in Those with Stage I Parkinson's Disease Utilizing LSVT BIG®

**DOI:** 10.1155/2017/9871070

**Published:** 2017-02-26

**Authors:** Beth Millage, Erin Vesey, Marsha Finkelstein, Mattie Anheluk

**Affiliations:** ^1^Courage Kenny Rehabilitation Institute, Mercy Hospital, Allina Health, Minneapolis, MN, USA; ^2^Courage Kenny Research Center, Allina Health, Minneapolis, MN, USA; ^3^Courage Kenny Rehabilitation Institute, Abbott Northwestern, Allina Health, Minneapolis, MN, USA

## Abstract

Individuals with Parkinson's Disease (PD) are often not referred to Physical Therapy (PT) until there are issues with mobility in later Hoehn and Yahr Stages. There have been no studies outlining the benefits of PT intervention in Stage I only. For persons with PD, deficits in motor function increase over time due to destruction of dopamine-producing cells. LSVT BIG, an exercise program for PD, has been shown to be effective in improving mobility. The purpose of this study was to assess participants functional improvement at a level of minimal clinically important difference (MCID) in one of four outcome measures: Gait Speed, Berg Balance Assessment, Functional Gait Assessment, and Unified Parkinson's Disease Rating Scale Motor Section.* Case Description.* Nine participants with Stage I PD received LSVT BIG 4x/week for 4 weeks followed by bimonthly participation in a community class. Outcome measurement occurred at baseline, after LSVT BIG, and three months after LSVT BIG.* Outcomes.* Eight of nine participants (88.9%) achieved MCID in at least one of the four measures at both after and 3 months after LSVT BIG training indicating improvement based on our criteria. Participants in Stage I of PD in this study completed LSVT BIG and demonstrated improved function.

## 1. Introduction

For persons diagnosed with Parkinson's Disease (PD), deficits in motor function increase during the course of the disease, intensifying disability. These deficits are the result of loss of dopamine-secreting neurons in the motor circuits of the basal ganglia which are essential for control and coordination of movement [1]. This degeneration manifests as the cardinal motor features of PD: bradykinesia, resting tremor, rigidity of muscles, and impaired posture [2]. There is evidence that exercise is neuroprotective and, if done regularly, can help the brain produce growth factors to protect dopamine-producing neurons from early death [3]. Animal studies have indicated that increased use of a limb can reduce neurotoxins, thereby preserving dopamine neurons, and potentially slowing or temporarily halting progression of motor deficits related to PD [4]. This is important for those in early Hoehn and Yahr Stages of PD where unilateral involvement can cause functional limitations and asymmetrical movements or compensatory movement strategies.

A comprehensive exercise approach in treating PD used in Physical and Occupational Therapy is the “Lee Silverman Voice Treatment (LSVT) BIG”. This approach was developed and evaluated by Drs. Becky Farley and Gail Koshland. It began as a concept of “Learning and Training BIG” based on principles first described and researched for LSVT LOUD®, where the primary treatment focus was amplitude [5]. Individuals with PD tend to overestimate their ability to complete tasks with the proper amplitude, inaccurately perceiving their movements to be appropriate in size and strength. They often do not step, reach, or walk far enough without visual or verbal feedback [5, 6]. LSVT BIG is designed to treat specific symptoms related to these deficits in movement patterns including bradykinesia or akinesia, decreased postural control and awareness, decreased gait mechanics and stability, and decreased balance [5]. The goal is to teach participants to carry over and sustain bigger movements in their daily activities [6]. The effect of BIG is achieved by targeting damaged basal ganglia through repetitive activation across motor regions in the brain that are involved in normal amplitude movements [3, 5, 7]. An effort scale helps participants learn to calibrate their movements to overcome the sensory mismatch between perceived movement and the actual completed movement. The LSVT BIG approach is unique in incorporating shaping techniques through use of therapist modeling or tactile/visual cues, improving self-perception and leading to improvement in movement patterns [1].

LSVT BIG has been shown to be effective in improving mobility for people with PD in a variety of stages of the disease [1, 6, 12]. There are have been studies that have investigated LSVT BIG and the effectiveness, but all are focused on multiple Hoehn and Yahr Stages of PD. Most research articles focus on Hoehn and Yahr Stages I–III with no studies focused solely on Stage I [12]. These individuals are not often referred for PT interventions until they or their care partner note severe issues with mobility [5]. Therapy referrals are often reactive rather than proactive in managing secondary impairments of PD. For example, referrals may occur when individuals develop postural instability and decreased muscle strength which leads to falls and increased risk of morbidity and mortality [13]. Drug therapies and surgical interventions have been shown to provide symptomatic relief; however, even with these interventions, motor deficits continue to progress during the course of the disease [6]. Exercise has been previously established in a number of studies to be an adjunct therapy to medication, which may provide even more benefit. Previous studies that included participants in Hoehn and Yahr Stages I to III found that improvements in amplitude and speed were greatest in Stage I for reaching and gait [12]. Those in Stage I do not always complete normal movement patterns, leading to early nonuse and further degeneration, implying that individuals in Stage I can potentially realize significant gains from BIG training [12].

The primary purpose of this study was to explore how LSVT BIG can impact individuals in Stage I of PD. Care providers need evidence that referral at early stages to a PT program such as LSVT BIG is beneficial [8, 14]. Currently, there are no known studies that focus exclusively on this subset of PD patients. A secondary objective was to explore adherence to exercise recommendations to better assist in prescribing exercise that can be completed through the lifespan.

### 1.1. Discussion

This study was conducted at a hospital-based outpatient neurological rehabilitation clinic. Participants were referred to Physical Therapy from local neurologists and primary care physician clinics. Patients were eligible to participate if they were in Stage I of clinically probable idiopathic PD, could communicate in English, able to attend a community LSVT BIG exercise class, and willing to sign consent [15]. Exclusion criteria included inability to participate in exercise due to comorbid conditions or having had Deep Brain Stimulation.  Table 1 describes patient demographics and other characteristics.

Eleven people presented with PD with symptoms of unilateral bradykinesia with hypokinesia. All potential participants reported changes in gait, balance, and decreased quality of life. Initial patient interview indicated they were in Stage I and found to be eligible for the study. Of those, nine provided written consent to participate in this study.

The examination completed by the Physical Therapist certified in LSVT BIG consisted of a semistructured interview and physical examination. Participants completed a questionnaire regarding their past medical history, current symptoms and goals, and past interventions (of note, no participants reported past PT for their PD symptoms). Subjective history was taken which included information about diagnosis, medication, social support, and functional limitations to be addressed in LSVT BIG intervention.  Table 2 outlines the clinical characteristics of each participant at evaluation. Objective measures included primary and secondary outcomes.

### 1.2. Outcome Measures

The objective tests and measures confirmed all participants were in Stage I of PD and indicated they were appropriate and would benefit from LSVT BIG intervention. Tables 4 and 5 outline their baseline scores on the primary and secondary outcomes measures which were used to determine this information. Follow-up testing of all objective measurements was to be completed after BIG training and 3 months after BIG training. Phone calls were also to be utilized throughout the intervention (every two weeks) to monitor adherence to the home exercise program.

#### 1.2.1. Gait and Balance Measures

The objective measures for gait and balance assessment included the primary outcomes of Gait Speed, Berg Balance Assessment, and Functional Gait Assessment (FGA), as well as a secondary outcome, the Four-Square Step Test (FSST). These were designated based on availability of MCID criteria.  Table 3 describes criteria for minimal clinically important difference (MCID) and Minimal Detectable Change (MDC).

Gait Speed is calculated as time required to complete a 30-foot walk and can help quantify bradykinesia with ambulation in PD [8]. The Berg Balance Assessment, a measure of balance in the older adult, is a 14-item scale [9]. In PD, this has excellent test-retest reliability (ICC = 0.80) and interrater reliability (ICC = 0.95) [9]. The validity of the Berg is also excellent with high predictive ability for falling as compared to other balance assessments in PD [16]. The FGA, a 10-item scale used to assess motor function and predict falls, has high test-retest reliability of 0.91 [17]. For people with PD, a cut-off score of 15/30 was found to be predictive of falls (sensitivity = 0.72 and specificity = 0.78) [17]. The FSST is measured by timing a participant's ability to step in multiple directions. The interrater reliability for this test is high (0.99) as well as the test-retest reliability (ICC = 0.78) [18].

#### 1.2.2. PD Symptoms and Quality of Life Measures

The objective measures for this category included the primary outcome, the Unified Parkinson's Disease Rating Scale III Motor Section (UPDRS), and the secondary outcome, the Parkinson's Disease Questionnaire-39 (PDQ-39). These were based on availability of MCID criteria. The UPDRS Motor is a 14-item scale and is considered the “gold-standard” for experimental studies and medical management [19]. This tool is also useful for general staging of PD relative to function, disability, and monitoring change in participant status throughout the course of treatment and over episodes of care [10]. The PDQ-39, the most widely used disease specific self-assessment rating tool in PD [20], contains eight domains (mobility, activities of daily living, emotional well-being, stigma, social support, cognition, communication, and bodily discomfort) [11, 20]. We report on results of the mobility domain specifically, given the nature of the LSVT BIG intervention. The mobility domain has a high correlation with the Hoehn and Yahr Index (*r* = 0.63) indicating this measure may also help with stage determination [21]. The PDQ-39 has high test-retest reliability (0.68–0.95) (ICC = 0.55) [21].

#### 1.2.3. PD Knowledge Survey

At baseline, participants were asked to complete a brief four question knowledge survey about PD to determine awareness of the disease. Three of the questions were derived from an article from Valldeoriola et al. with the addition of the fourth question by the investigators [22]. The questions included the following: (1) Is PD a chronic disease? (2) Can PD be reasonably controlled by medication? (3) Do you know at least two symptoms related to the disease? (4) Can symptoms of PD be controlled with exercise? The fourth question was used to determine participants' understanding of the benefits of exercise in PD. Results provided the treating therapist with a clearer picture of the participant's educational needs about PD. See Table 1 for results.

### 1.3. Intervention

#### 1.3.1. LSVT BIG Exercise Program

The participants attended a total of 16 sessions of one-on-one training with a therapist certified in the LSVT BIG approach and completed the intervention per the protocol. Sessions were one hour each, four times per week for four weeks [3, 6, 7]. Exercises combine standing and sitting movement patterns to create larger-amplitude movements and improve movement initiation and motor control. These exercises also are sustained, multidirectional, and functional movements (Table 6). The program is designed to be intensive, high effort, and complex with many repetitions to maximize practice and carryover to functional tasks [3, 6]. This training is consistent with principles that promote activity-dependent neuroplasticity for specificity, intensity, repetition, and saliency [4, 5]. A BIGness Effort scale (range 0 to 10) from LSVT Global®, based on a modified Borg Rating of Perceived Exertion, was utilized at each session [23]. Participants were instructed to complete each exercise at an effort rating of seven or greater on this scale in order to achieve a reasonably high level of exertion. In addition to the exercises, there was a focus on “carryover assignments” as a method to address functional limitations in the home environment. “Hierarchy tasks” are functional tasks involving a series of movements and were utilized to address sequential movement patterns to develop strategies to achieve a functional goal. These activities were designed to help the participant generalize the activities learned in PT sessions and apply them in daily life. Participants received a binder, the “LSVT BIG Survival Guide” which included materials from LSVT Global for an introduction to the principles of LSVT BIG, the BIGness Effort scale, a home exercise program log, and copies of the protocol exercises (picture and text format for each exercise). Informational materials for the participant and family about PD and local PD support groups were also compiled by the investigators and issued to each participant.

#### 1.3.2. Adherence Intervention

Participants were contacted by telephone every other week over the study period of four months. They were asked questions to explore their adherence to the recommended home exercise program. Barriers were discussed and participants were directed to talk with the Principal Investigators (PIs) when suboptimal adherence was noted. Participants were asked to track their adherence with exercise at home using a checklist throughout the study.

#### 1.3.3. Community Exercise Class

Participants were required to attend an LSVT BIG Community Exercise Class for three months, twice a month, for a total of six classes, following completion of LSVT BIG training. The classes consisted of LSVT BIG exercises, functional training, strength exercises, and balance activities. Attendance at these sessions was recorded.

### 1.4. Outcome

Participants were successful with LSVT BIG if they achieved minimal clinically important difference (MCID) in functional improvement on at least one of four primary outcome measures: Gait Speed, Berg Balance Assessment, FGA, or UPDRS (Table 3). Summary statistics including median (minimum, maximum) and total number are used to describe the population and outcomes. For the PDQ-39, Minimal Detectable Change (MDC) was used to evaluate each participant's change from baseline in place of MCID since it is currently not defined [11].

Tables 4 and 5 describe the primary and secondary outcomes, providing baseline values and changes from baseline, after LSVT BIG training (delta post), and 3 months after LSVT BIG training (delta 3 months). Eight of nine participants achieved functional improvement of MCID in at least one of the four primary measures at both after and 3 months after LSVT BIG training. Participant 8 did not meet criteria for success as he was functioning at a very high level and therefore was limited in possible range of improvement with ceiling effect noted in some measures. With the exception of Gait Speed, Participants 3, 5, 8, and 9 were in this same situation on one or more of the primary measures. Six participants (1, 3, 4, 6, 7, and 8) met the MDC improvement criteria for the PDQ-39 at both after and 3 months after training. Participants 2 and 9 had motor domain scores that were <8 at baseline and therefore it was not possible to achieve improvement at MDC of 12.24. When comparing post-BIG training and 3 months after BIG training, there was little change in performance of the four primary measures.

Over the initial four week period of training, data obtained through exercise adherence checklists indicated that the number of days missed for completion of home exercises was three or less. Participants 3 and 8 sustained minor injury unrelated to the study, and as a result they modified their exercises for one week. Participants exercised once instead of the recommended twice daily schedule on a few occasions. Seven of nine participants attended all community classes. Participant 2 attended five classes, and Participant 8 was unable to attend any classes due to conflicts with work.

Adherence was further explored through semistructured telephone interviews. Data obtained through telephone interviews during the initial four week period of training supported participants' reports of adherence. Participants 1, 2, 3, 5, and 9 reported adherence to program as instructed. Participants 4 and 6 were adherent to exercises at a varied schedule of completing double the amount of exercises, one time a day. Participant 7 reported completing one set of exercises daily instead of two due to reported foot pain. Participant 8 was generally nonadherent during the initial training, reporting completing weekends only due to other life demands. Overall, impressions of adherence obtained through telephone interviews suggest adherence rates generally declined after the initial four weeks of exercise training.

After the initial four weeks, telephone interviews continued for the remainder of the study. Participant 2 was the only one who consistently reported completing exercises as recommended two times daily. Six participants (3, 4, 5, 6, 7, and 9) reported adherence at a varied schedule such as once a day, doubling or spacing exercises out throughout the day, or missing occasional days or weeks. Participant 8 reported variable adherence due to life demands and Parkinson's symptoms.

## 2. Discussion

In this study, the participants benefited from LSVT BIG as shown by improvements on at least one of the primary outcomes measures at a level of MCID: Gait Speed, Berg Balance Assessment, FGA, and/or UPDRS. These improvements were achieved after LSVT BIG training and were maintained at three months after training for 8 of 9 participants. The participants in this study reported improvements in quality of life as outlined in the PDQ-39. Their baseline reporting of symptoms and limitations was more significant than researchers originally anticipated for people who are in Stage I PD. Continued research of Stage I PD is urgent given that approximately 1.5 million people in the United States have PD and with the extension of life expectancy, the number of individuals with PD is expected to rise dramatically within the next twenty-five years [24]. Even 10% slowing of the disease would allow individuals to maintain an improved and more productive quality of life and also may yield significant savings in healthcare costs [3].

We required attendance at a community class as a means of reinforcement of LSVT BIG training as well as encouraging adherence to a daily exercise routine. Our results at three months showed little or no loss in performance from the advances made after LSVT BIG training. Group-based exercise through community classes has been shown to increase quality of life for neurological conditions, especially PD [25, 26]. Attendance can influence longer-term participation in exercise where one study reported that 44% of participants continued to exercise after the community program ended [25]. Additional benefits include facilitating exercise in a safe and social environment, providing a consistent exercise schedule, and a sense of accountability that holds group members to participate [26].

Reliable methods for supporting adherence to home exercise programs are needed to help participants be successful in adopting a regular exercise routine and schedule. Participants were contacted every other week by phone and were encouraged to identify barriers to exercising routinely. Those who identified barriers were provided guidance in overcoming them. Additional research is needed to explore factors associated with PD and exercise adherence including elements such as barriers and facilitators to exercise adherence, exercise preferences, self-efficacy, exercise attitudes, and motivation. Research exploring strategies that clinicians can use to promote development of habits and routines related to exercise is also needed. Clinicians need to consider the complexities of adherence when providing recommendations [27].

Many participants come to Physical Therapy with limited knowledge on how effective exercise can be in treating their condition. Based on the knowledge survey administered as part of this study, Participants 1 and 3 did not think that exercise could help symptoms of PD. This may be due to lack of information that is available to people newly diagnosed with PD with regard to the role of exercise throughout the disease process. As stated previously, most participants are not referred for PT until they are experiencing falls or have a significant change in their functional ability. Educating care providers about potential role of exercise in PD is important in raising awareness to ameliorate symptoms and improve quality of life [28].

### 2.1. Limitations

In this study, both the Principal Investigator and Coinvestigator, who are both Physical Therapists, delivered the LSVT BIG training. Since LSVT BIG training has a prescribed methodology and credentialing requirements, we are confident that participants received training as prescribed. However, their teaching techniques may have had some variations that could have affected learning and retention.

We did not screen for deficits in cognition prior to participation in this study, which may be an additional limitation [18]. Past research has shown that a large number of people with PD have associated dementia with cognitive changes sometimes occurring early in the disease process, most prominently with memory and executive function [29]. This factor could influence follow-through with PT interventions and recommendations as well as the ability to learn and adhere to home program.

Another limitation to this study was that Participant 8 was an outlier, but the researchers felt it was important to include his data to examine tests and measures that may have a ceiling effect in Stage I of PD. He was also included in the study results even though he had varied adherence to home program and community classes due to full-time work and other life demands with raising children. This can be a limitation with research and outcomes with Stage I PD as individuals are often more active in community and family roles compared with those in later stages of the disease.

At the time of this research study, there was little research related to the best evidence and tests for balance and gait in PD. For this reason, the Berg was chosen to assess balance because there was normative data for people with PD. Since that time, there has been further research regarding more specific tests and measures that are appropriate for multiple stages of PD by the American Physical Therapy Association. For example, this has led to further use of the Mini-BEST test in PD. Future research would be beneficial in determining usefulness in Stage I of PD including normative data.

### 2.2. Implications for Future Research

Additional research is needed to show efficacy in LSVT BIG training in Stage I of PD, to explore adherence to exercise in this population and whether routine exercise can slow disease progression.

## Figures and Tables

**Table 1:** Participant demographics.

Characteristic	Statistic
Age (years), median (range)	75 (51–82)
Sex	
Female	5
Male	4
Race	
White	9
Care partner available at home	4
# comorbidities, median (range)	5 (0–11)
# medications, median (range)	6 (2–17)
# PD medications, median (range)^*∗*^	1 (0–2)
0	1
1	5
2	3
# dosing times/24 hours	
2	2
3	6
4	1

^*∗*^Medication details: *participant 1: carbidopa/levodopa and rasagiline, participants 2, 4, 5, and 7: carbidopa/levodopa, participant 3: carbidopa/levodopa and ropinirole, participant 6: none, participant 8: carbidopa/levodopa and cyclobenzaprine, and participant 9: pramipexole*.

**Table 2:** Clinical characteristics of participants.

ID	Chief complaints	Comorbidities	Patient goal	Time from diagnosis to start of LSVT BIG
1	Muscle Stiffness, difficulty with walking and stairs, difficulty with upper extremity tasks	Irritable bowel syndrome, gastric reflux, hypertension, hyperlipidemia, osteopenia, skin cancer, urge incontinence, chronic obstructive pulmonary disease	“To improve symptoms, walk better, relax muscles”	2 months
2	Poor posture, difficulty walking, decreased balance	Hypertension, obesity, low back pain, osteopenia, macular degeneration, bilateral rotator cuff surgery	“Improve walking and decrease symptoms”	11 months
3	Fatigue, difficulty with fine motor skills, left upper extremity tremor	Depression	“Improve endurance of left arm, increase exercise”	40 months
4	Difficulty with transfers and gait, freezing, left side tremor	Glaucoma, hypertension, left ankle surgery, hypothyroidism, cataracts	“Improved movement and increased strength”	13 months
5	Lower extremity weakness, difficulty with gait	Prolactinoma	“Restore strength and flexibility”	17 months
6	Slow Gait Speed, unsteady gait	Diabetes mellitus, diastolic heart failure, hypertension, atrial fibrillation, small bowel resection, hyperlipidemia, left knee and shoulder arthroscopy, lumbar surgery, total knee arthroplasty, iridotomy	“Improve balance and function”	29 months
7	Neck and foot pain, fearful of falling	Bilateral total knee arthroplasty	“Get muscles stronger to get more balanced”	3 months
8	Decreased arm swing, scapular pain, rigidity	None	“Loosen trap muscles and improve symptoms of PD”	65 months
9	Sit to stand, buttoning shirt, poor handwriting	Hypertension, cardiac stent, endarterectomy, constipation, bladder issues, difficulty in hearing	“Learn how to do things better”	53 months

**Table 3:** Outcome measures (completed at baseline, after LSVT BIG, and 3 months after LSVT BIG).

Measure	Range	MCID or MDC
Gait Speed (meters/sec)	NA	0.16 (MCID) [8]
Berg Balance Assessment	0–56	4 points if initial score is 45–56, 5 points if the initial score is 35–44, 7 points if the initial score is 25–34, 5 points if the initial score is 0–24 (MCID) [9]
FGA	0–30	8 (MCID)
UPDRS, Motor Section	0–56	−2.5 points (MCID) [10]
Four-Square Step Test (secs)	NA	Not established at time of study
PDQ-39, Mobility Domain	0–156	−12.24 (MDC) [11]
Knowledge Survey (baseline only)	0–5	Not applicable

**Table 4:** Primary outcomes and MCID.

ID	Gait Speed Baseline, delta post, delta 3 mths	FGA Baseline, delta post, delta 3 mths	Berg Baseline, delta post, delta 3 mths	UPDRS Baseline, delta post, delta 3 mths	Total MCID Post, 3 mths
1	0.99, 0.19^*∗*^, 0.27^*∗*^	19, 7, 11^*∗*^	52, 4^*∗*^, 4^*∗*^	7, −3^*∗*^, −6^*∗*^	3, 4
2	0.95, 0.10, 0.24^*∗*^	18, 3, 7	50, 1, 3	11, −8^*∗*^, −4^*∗*^	1, 2
3	1.11, 0.62^*∗*^, 0.60^*∗*^	27, 3, 3^1^	53, 3, 3^2^	10, −6^*∗*^, −5^*∗*^	2, 2
4	1.0, 0.10, 0.19^*∗*^	11, 14^*∗*^, 13^*∗*^	34, 16^*∗*^, 18^*∗*^	30, −24^*∗*^, −24^*∗*^	3, 4
5	1.27, 0.30^*∗*^, 0.10	22, 5, 7	55, 1, 1^2^	16, −7^*∗*^, −3^*∗*^	2, 1
6	1.2, −0.28, −0.02	19, 4, 1	46, 6^*∗*^, 7^*∗*^	10, −1, 3	1, 1
7	0.76, 0.18^*∗*^, 0.09	10, 11^*∗*^, 8^*∗*^	35, 14^*∗*^, 16^*∗*^	25, −18^*∗*^, −20^*∗*^	4, 3
8	1.4, −0.01, 0.10	30, 0, 0^1^	55, 0, −2^2^	2, 1, 0^3^	0, 0^4^
9	1.02, 0.14, 0.47^*∗*^	16, 9^*∗*^, 7	53, 1, −3^2^	28, −17^*∗*^, −19^*∗*^	2, 2
Total MCID	Post: 4	Post: 3	Post: 4	Post: 7	8 participants achieved MCID in at least 1 measure
	3 mths: 5	3 mths: 3	3 mths: 4	3 mths: 7	

^*∗*^Meeting MCID required improvement in performance from baseline.

^1^For Functional Gait Assessment, 2 of 9 participants' baseline was so high that improvement at a level of MCID was not possible. In fact, participant 8 was at the maximum score possible (=30) at baseline.

^2^For Berg, 4 of 9 participants' baseline was so high that improvement at a level of MCID was not possible.

^3^For UPDRS, 1 participant's baseline was so low that improvement at a level of MCID was not possible.

^4^Participant 8 scored well on functional assessments at baseline on the FGA, Berg, andUPDRS, so improvement at a level of MCID was not possible to achieve.

**Table 5:** Secondary outcomes.

ID	Four-Square Step Clockwise Baseline, delta post, delta 3 mths	Four-Square Step Counter*-*Clockwise Baseline, delta post, delta 3 mths	PDQ-9, Mobility Baseline, delta post, delta 3 mths	Knowledge survey Total score (range 0–5)
1	3.3, −0.17^*∗*^, 0.08	3.5, −0.2^*∗*^, 0.03	25, −7.5, −22.5^*∗*^	4^2^
2	5.1, 0.3, 1.1	5.3, −0.4^*∗*^, 0.6	7.5, 22.5, 35^1^	5
3	3.1, −0.2^*∗*^, −0.7^*∗*^	2.8, 0, −0.5^*∗*^	40, −35^*∗*^, −37.5^*∗*^	4^2^
4	9, −4.1^*∗*^, −4.8	8.1, −2.0^*∗*^, −3.5^*∗*^	85, −50^*∗*^, −35^*∗*^	5
5	5.9, −1.0^*∗*^, −0.9^*∗*^	6.1, −1^*∗*^, −0.7^*∗*^	17.5, 5, −5	5
6	7.3, −1.1^*∗*^, 1	7.1, 1.3, −1.0^*∗*^	32.5, −20^*∗*^, −17.5^*∗*^	4
7	12.5, −7.8^*∗*^, −6.4^*∗*^	12.3, −7.7^*∗*^, −5.5^*∗*^	62.5, −17.5^*∗*^, 10	5
8	4.2, −1.3^*∗*^, −0.7^*∗*^	4.09, −1.2^*∗*^, −0.8^*∗*^	20, −20^*∗*^, −20^*∗*^	5
9	6.1, −0.3^*∗*^, −2.5^*∗*^	5.6, −0.8^*∗*^, −1.1^*∗*^	5, 0, −5^1^	5

	Total Improved	Total Improved	Total MDC	
	Post: 8	Post: 7	Post: 5	
	3 mths: 6	3 mths: 7	3 mths: 5	

^*∗*^Four-Square Step indicates improvement from baseline, PDQ-9 Mobility indicates meeting MDC required improvement in performance from baseline.

^1^These participants had such low scores that improvement at a level of MDC was not possible.

^2^These participants did not believe that exercise could help alleviate symptoms of PD.

**Table 6:** LSVT BIG interventions described.

Task	Exercises	Progression
Maximal sustained exercises	Exercise 1: 8 repetitions floor to ceiling Exercise 2: 8 repetitions side to side	Finger weights, flicks

Repetitive standing movements	Exercise 3: 16 repetitions forward step Exercise 4: 16 repetitions sideways step Exercise 5: 16 repetitions backward step Exercise 6: 20 repetitions rock and reach forward Exercise 7: 20 repetitions reach and twist sideways	Starting with external support chair working towards no assistive device, adding cuff weights to limbs, larger weight shifts

Functional components and hierarchy task	All did sit/stand and then 4 other functional components selected based on chief complaints	Adding cuff weights to limbs, reducing cues and shaping, increasing reps, increasing speed, changing support surface dynamics

Hierarchy task	A sequence of functional components	Increasing reps, increasing speed, adding more complexity with props or functional items for the task

BIG walking/gait training	Distance varied from 110′ to 825′, focus on longer strides with reciprocal arm swing and upright posture	Decrease use of AD, longer distances, outdoor surfaces, hiking poles, metronome, treadmill training, and reciprocal arm swing

## References

[1] Janssens J., Malfroid K., Nyffeler T., Bohlhalter S., Vanbellingen T. (2014). Application of LSVT BIG intervention to address gait, balance, bed mobility, and dexterity in people with Parkinson disease: a case series. *Physical Therapy*.

[2] Bohlhalter S., Kaegi G. (2011). Parkinsonism: heterogeneity of a common neurological syndrome. *Swiss Medical Weekly*.

[3] Farley B. G., Fox C. M., Ramig L. O., McFarland D. H. (2008). Intensive amplitude-specific therapeutic approaches for Parkinson's disease: toward a neuroplasticity-principled rehabilitation model. *Topics in Geriatric Rehabilitation*.

[4] Kleim J. A., Jones T. A., Schallert T. (2003). Motor enrichment and the induction of plasticity before or after brain injury. *Neurochemical Research*.

[5] Fox C., Ebersbach G., Ramig L., Sapir S. (2012). LSVT LOUD and LSVT BIG: behavioral treatment programs for speech and body movement in Parkinson disease. *Parkinson's Disease*.

[6] Ebersbach G., Ebersbach A., Edler D. (2010). Comparing exercise in Parkinson's disease—the Berlin LSVT BIG study. *Movement Disorders*.

[7] Ebersbach G., Ebersbach A., Gandor F., Wegner B., Wissel J., Kupsch A. (2014). Impact of physical exercise on reaction time in patients with Parkinson's disease—data from the berlin BIG study. *Archives of Physical Medicine and Rehabilitation*.

[8] Perera S., Mody S. H., Woodman R. C., Studenski S. A. (2006). Meaningful change and responsiveness in common physical performance measures in older adults. *Journal of the American Geriatrics Society*.

[9] Donoghue D., Stokes EK. (2009). How much change is true change? The minimum detectable change of the Berg Balance Scale in elderly people. *Journal of Rehabilitation Medicine*.

[10] Shulman L. M., Gruber-Baldini A. L., Anderson K. E., Fishman P. S., Reich S. G., Weiner W. J. (2010). The clinically important difference on the unified Parkinson's disease rating scale. *Archives of Neurology*.

[11] Fitzpatrick R., Norquist J. M., Jenkinson C. (2004). Distribution-based criteria for change in health-related quality of life in Parkinson's disease. *Journal of Clinical Epidemiology*.

[12] Farley B. G., Koshland G. F. (2005). Training BIG to move faster: the application of the speed-amplitude relation as a rehabilitation strategy for people with Parkinson's disease. *Experimental Brain Research*.

[13] Ashburn A., Stack E., Pickering R. M., Ward C. D. (2001). A community‐dwelling sample of people with Parkinson's disease: characteristics of fallers and non‐fallers. *Age and Ageing*.

[14] Pentecost C., Taket A. (2011). Understanding exercise uptake and adherence for people with chronic conditions: a new model demonstrating the importance of exercise identity, benefits of attending and support. *Health Education Research*.

[15] Gelb D. J., Oliver E., Gilman S. (1999). Diagnostic criteria for Parkinson disease. *Archives of Neurology*.

[16] Brusse K. J., Zimdars S., Zalewski K. R., Steffen T. M. (2005). Testing functional performance in people with Parkinson disease. *Physical Therapy*.

[17] Leddy A. L., Crowner B. E., Earhart G. M. (2011). Functional gait assessment and balance evaluation system test: reliability, validity, sensitivity, and specificity for identifying individuals with parkinson disease who fall. *Physical Therapy*.

[18] Duncan R. P., Earhart G. M. (2013). Four Square Step Test performance in people with parkinson disease. *Journal of Neurologic Physical Therapy*.

[19] Goetz C. C., Poewe W., Rascol O. (2003). The Unified Parkinson's Disease Rating Scale (UPDRS): status and recommendations. *Movement Disorders*.

[20] Hagell P., Nygren C. (2007). The 39 item Parkinson's disease questionnaire (PDQ-39) revisited: implications for evidence based medicine. *Journal of Neurology, Neurosurgery and Psychiatry*.

[21] Peto V., Jenkinson C., Fitzpatrick R., Greenhall R. (1995). The development and validation of a short measure of functioning and well being for individuals with Parkinson's disease. *Quality of Life Research*.

[22] Valldeoriola F., Coronell C., Pont C. (2011). Socio-demographic and clinical factors influencing the adherence to treatment in Parkinson's disease: the ADHESON study. *European Journal of Neurology*.

[23] Borg G. A. V. (1982). Psychophysical bases of perceived exertion. *Medicine and Science in Sports and Exercise*.

[24] Abdullah R., Basak I., Patil K. S., Alves G., Larsen J. P., Møller S. G. (2015). Parkinson's disease and age: the obvious but largely unexplored link. *Experimental Gerontology*.

[25] Ploughman M., Shears J., Harris C. (2014). Effectiveness of a novel community exercise transition program for people with moderate to severe neurological disabilities. *NeuroRehabilitation*.

[26] States R. A., Spierer D. K., Salem Y. (2011). Long-term group exercise for people with Parkinson's disease: a feasibility study. *Journal of Neurologic Physical Therapy*.

[27] Radomski M. V. (2011). More than good intentions: advancing adherence to therapy recommendations. *American Journal of Occupational Therapy*.

[28] Kremens D., Hauser R. A., Dorsey E. R. (2014). An update on Parkinson's disease: improving patient outcomes. *The American Journal of Medicine*.

[29] Hu M. T. M., Szewczyk-Królikowski K., Tomlinson P. (2014). Predictors of cognitive impairment in an early stage Parkinson's disease cohort. *Movement Disorders*.

